# Validation of the prophylactic efficacy of urea‐based creams on sorafenib‐induced hand‐foot skin reaction in patients with advanced hepatocellular carcinoma: A randomised experiment study

**DOI:** 10.1002/cnr2.1532

**Published:** 2021-12-14

**Authors:** Ru‐Yu Lien, Heng‐Hsin Tung, Shang‐Laing Wu, Sophia H. Hu, Ling‐Chun Lu, Shu‐Fen Lu

**Affiliations:** ^1^ Department of Nursing Taipei Veterans General Hospital Taipei Taiwan; ^2^ Department of Nursing National Yang Ming Chiao Tung University Taipei Taiwan; ^3^ Biostatistical Consultant, School of Medicine Griffith University Gold Coast Australia; ^4^ Second Degree of Science in Nursing, College of Medicine National Taiwan University Taipei Taiwan; ^5^ National Taiwan University Hospital Yunlin Branch Yunlin Taiwan

**Keywords:** head‐foot skin reaction, hepatocellular carcinoma, side effect of Sorafenib, symptom management

## Abstract

**Background:**

Hand‐foot skin reaction may influence the effectiveness of patients' treatment, patient quality of life, and the economics of health care. An effective prophylactic dermatological cream for preventing sorafenib‐induced hand‐foot skin reaction (HFSR) is yet to be identified.

**Aim:**

The aim of this study is validated the prophylactic efficacy of urea‐based creams on sorafenib‐induced hand‐foot skin reaction in patients with advanced hepatocellular carcinoma.

**Methods:**

This was a randomised double‐blind experimental study. A total of 129 patients with advanced HCC were randomly assigned to three groups. The comparison group received best supportive care (BSC), group A received BSC plus a moisturising cream, and group B received BSC plus a 10% urea‐based cream. Incidence of HFSR and cutaneous wetness were assessed 3 days before starting sorafenib and each week after starting sorafenib for 8 weeks.

**Results:**

No significant difference was observed in the incidence density of sorafenib‐induced HFSK (comparison group/A group, *p* > .05; comparison group/B group, *p* > .05). Group B reported significantly better cutaneous wetness of hands in the seventh week after starting sorafenib (*p* < .05) and of feet during the first 6 weeks (*p* < .05–.001).

**Conclusion:**

This study found a nut size amount of a 10% urea‐based cream applied twice a day can maintain patients' cutaneous wetness in the first 6 weeks after starting sorafenib than moisturising‐alone cream. But it cannot reduce the occurrence of HFSR. Thus, the result supports nut‐size dose of the 10% urea‐based cream three times a day may be an appropriate dose to prevent HFSR.

Clinical Trail Registration Number: NCT04568330.

## 1 INTRODUCTION

Cancer incidence and mortality worldwide are increasing rapidly and are responsible for the majority of global deaths.^1^, ^2^, ^3^ According to estimates by GLOBOCAN 2018 from the International Agency for Research on Cancer in the World Health Organisation, liver cancer is the fifth most commonly diagnosed cancer and the fourth leading cause of cancer death, with 841 080 new diagnoses and 81 631 cancer deaths.^2^ Of the pathological types, 85%–90% are hepatocellular carcinoma (HCC), the highest incidence of which is in East Asia.^1^, ^2^, ^3^, ^4^

The Sorafenib Hepatocellular Carcinoma Assessment Randomised Protocol (SHARP) demonstrated that sorafenib offers significant benefits in terms of prolonging median overall survival from 7.9 to 10.7 months (hazard ratio in the sorafenib group, 0.69; 95% confidence interval [CI], 0.55–0.87; *p* < .001) and radiologic progression from 2.8 to 5.5 months (hazard ratio in the sorafenib group, 0.58; 95% CI, 0.45–0.74; *p* < .001).^5^ In the Asia‐Pacific population with advanced HCC and Child–Pugh liver function class A, it prolonged median overall survival from 4.2 to 6.5 months (hazard ratio in the sorafenib group, 0.68; 95% CI, 0. 50–0.93; *p* < .05) and median time to progression from 1.4 to 2.8 months (hazard ratio in the sorafenib group, 0.57; 95% CI, 0. 42–0.79; *p* < .001).^6^ Thus, sorafenib has been recommended as a standard treatment for advanced HCC.^3^, ^4^, ^5^, ^6^, ^7^

Sorafenib is an oral multikinase inhibitor that can block the proliferation of tumour cells by targeting the Raf/MEK/ERK signalling pathway.^8^ It also exerts an antiangiogenic effect by targeting the receptor tyrosine kinases vascular endothelial growth factor receptor (VEGFR)‐2, VEGFR‐3 and platelet‐derived growth factor receptor (PDGFR)‐β.^8^, ^9^ Approximately 10%–62% of patients treated with this agent reported a hand‐foot skin reaction (HFSR).^5^, ^6^, ^9^ HFSR is also called hand‐foot syndrome, palmer‐plantar erythrodysethesia, acral erythema, and Burgdorf reaction.^9^ It is a dose‐accumulated side effect, the highest incidence of which has occurred in patients in Asia‐Pacific undergoing advanced HCC treatment with sorafenib.^6^

The mechanisms of sorafenib‐induced HFSR remain unclear. One theory suggests that inhibitions of multikinase, VEGR, and PDGFR may impede vascular repair mechanisms and induce histopathological characteristics, including keratinocyte vacuolar degeneration, confluent keratinocyte necrosis, perivascular or lichenoid lymphocyte‐predominant infiltrate, spongiosis, hyperkeratosis, parakeratosis, and epidermal cleavage.^8^, ^9^, ^10^, ^11^ HFSR often occurs 2–6 weeks after starting sorafenib and is related to pharmacologic properties such as dose, peak plasma level, total accumulative dose, and schedule of administration.^8^, ^10^ The presentation of HFSR is unique with a prodrome comprising dysesthesia on the palms and soles with tingling.^9^ This then progresses to a burning pain and is followed by the symmetrical development of erythematous, edematous, and blistering plaques with hyperkeratosis.^9^, ^11^, ^12^ The HFSR can have a significant impact on patients' daily living activities, quality of life, and the economics of health care.^12^, ^13^

To date, varying forms of the management of sorafenib‐induced HFSR have been recommended based on grades of severity.^14^, ^15^ These grades are determined by the National Cancer Institute Terminology Criteria for Adverse Event (NCI‐CTCAE).^12^, ^14^, ^15^ During the pretreatment period, patients should be informed of dose‐dependent HFRS and asked to self‐report.^13^ Health care specialists must also comprehensively evaluate the baseline condition of patients' limbs.^12^, ^13^ A method of contacting health care specialists for the early diagnosis of HFSR should be available.^15^ Under grade I HFSR, patients may experience mild painless erythema, edema, hyperkeratosis, numbness, dysesthesia, paraesthesia, tingling, or swelling with no restriction of daily activity.^13^, ^14^ Suggested recommendations for management are to maintain the current dose of sorafenib, avoid hot water, use a moisturising cream and a 20%–40% urea‐based cream, and wear thick cotton gloves and socks.^13^, ^14^, ^15^ When patients develop grade II HFSR with painful peeling, blisters, bleeding, edema, erythema, or hyperkeratosis and their daily activity starts to become limited, a 50% dose reduction of sorafenib is recommended for 7–28 days along with treatment for grade I toxicity with an additional prescription of 0.05% clobetasol ointment and 2% lidocaine, codeine, or pregabalin.^14^, ^15^ Interruption of treatment and treatment for grade II HFSR before the severity improves to grade 0 or grade 1 are advised when grade III HFSR develops with symptoms that restrict daily activity, such as refractory painful moist desquamation, ulceration, blistering, or hyperkeratosis.^12^, ^15^

Urea can preserve cutaneous hydration and exert keratolytic action, therefore, urea‐based creams can improve the hyperkeratotic condition and has been widely used for tyrosine kinase inhibitor related HFSR.^14^, ^15^, ^16^ A randomised controlled trial validated the use of a 10% urea‐based cream with best supportive care (BSC) from treatment day 1 compared with BSC with no creams and reported significant prophylactic efficacy in terms of decreasing the grades of sorafenib‐induced HFSR in patients with advanced HCC (hazard ratio in the urea‐based cream group, 0.457; 95% CI, 0.344–0.608; *p* < .001) or delaying median time to first occurrence of HFSR (hazard ratio in the urea‐based cream group, 0.658; 95% CI, 0.541–0.799; *p* < .001).^17^ The use of an empiric 12.5% urea‐based cream for patients with advanced HCC and a hydrocolloid dressing containing ceramide on the feet of patients with renal cell carcinoma can improve sorafenib‐induced HRSF.^18^ This result indicates that the appropriate prophylactic dose of a urea‐based cream and its effectiveness compared with moisturising‐alone creams remain unclear.^17^, ^18^ The aim of this study was to validate the prophylactic HFSR incidence density and cutaneous wetness of 10% urea‐based creams on sorafenib‐induced HFSR in patients with advanced HCC.

## 2 METHODS

### 2.1 Design

**FIGURE 1:**
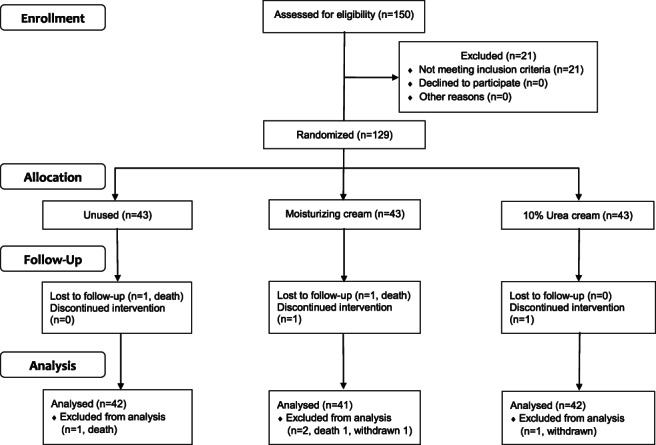
CONSORT diagram of patients included in the study

This was a randomised double‐blind experimental study. The sample size was estimated using G. power software version 3.1, which was established using logistic regression, odds ratio: 3.8, and power: 0.80.^19^ The estimated sample size needed to be at least 125. A CONSORT diagram of patients included in the study is presented in Figure 1. In total, 150 patients received sorafenib with HCC in a general medical centre in Taiwan between 1 January 2014 and 31 December 2014. Of these, 129 were eligible and agreed to participate. Using EXCEL random sampling, they were randomly assigned in a ratio of 1:1:1 to the following treatment groups: BSC alone (comparison group), BSC plus moisturising cream (group A), and BSC plus 10% urea‐based cream (group B). Each group initially comprised 43 patients; however, considering deaths and loss to follow‐up, four patients were eliminated from the study. Finally, a total of 42, 41, and 42 patients were included in the comparison group, group A, and group B, respectively. A case manager recruited the eligible patients and was also responsible for obtaining informed consent and providing patient education. A research employee was responsible for recording patients' demographic data, providing an unlabelled cream, and ensuring that the previous container of cream was not exhausted. A medical oncologist or a nurse was responsible for assessing the severity of HFSR and cutaneous wetness of patients. The assessment was conducted 3 days before starting sorafenib treatment and every 7 days after starting sorafenib treatment for 8 weeks. Creams were provided after the assessment. When patients developed HFSR, they were referred to the most appropriate form of management.

### 2.2 Inclusion and exclusion criteria

This study population comprised patients with the following characteristics: (1) HCC by proof of pathology; (2) the presence of a tumour thrombus in the main trunk of the portal vein or the first‐order branches of the portal vein with minimal or no ascetics by abdominal CT scan; (3) Child‐Pugh liver function class A; (4) plans to receive oral sorafenib 400 mg twice per day; (5) age of 20 years or above; and (6) ability to communicate in Chinese, Taiwanese, or Hakka. Patients with (1) encephalopathy, psychosis, cognition impairment, blindness, or a hearing impairment; (2) allergic history to urea; (3) ulceration, blisters, infective problems on the palms or soles; or (4) who had undergone previous surgery, systematic chemotherapy, or frequent radial ablation were excluded.

### 2.3 Intervention

The comparison group who received BSC alone were (1) informed of potential presentations of HFSR, (2) asked to wear waterproof gloves before undertaking household work or working with water, (3) provided with a method of contacting health care specialists for confirmation of an early diagnosis of HFSR, and (4) asked to self‐report when they experienced symptoms of HFSR. Group A received the same interventions as the comparison group but were additionally given the moisturising cream (dimethicone, fragrance free, Aveeno, United States) for use on nine occasions and were instructed how to use the cream. The instructions were to (1) use the cream twice a day from 3 days prior to starting sorafenib and each week thereafter, (2) scoop out a nut‐sized amount with a unique spoon each time, (3) gently apply the cream evenly on symmetrical palms below the wrists and symmetrical soles below the ankles each time, and (4) wear unique cotton gloves for 30 min immediately after application of the cream. Group B received interventions similar to those of group A, except that they were given a cream container with a different component (10% urea; Sipharr, Taiwan). The external appearance of the containers with the two kinds of cream was the same. The creams were white and grey in colour.

### 2.4 Outcomes and assessment

Data on confounders and two end‐points, incidence density of HFSR and cutaneous wetness, were collected. The confounders included gender, age, number of chronic illnesses, number of metastatic regions as well as levels of white count, haemoglobin, bleeding time, liver enzymes, albumin, and electrolyte.

The grades of HFSR were assessed using NCI‐CTCAE version 4. This is an available psychometric patient‐reported instrument developed by Dueck et al.^20^ Its test–retest reliability was 0.7 or greater for 36 of the 49 prespecified items (intraclass correlation coefficient, 0.76). Testing for divergent validity with the European Organisation for Research and Treatment of Cancer Core Quality of Life Questionnaire (OLO‐C30) showed a statistically significant correlation for 27 of the prespecified items (median *r* = .43, *p* < .05). An endpoint for incidence of HFSR is patients with grade 1 symptoms.

The level of cutaneous wetness was analysed using a skin scanner (SK‐03, Taiwan), which comprised bioelectrical‐impedance analysis (BIA) equipment. BIA is a safe, noninvasive, rapid, reproducible, portable, and inexpensive method of using simple equations for safely and accurately estimating the body water with a correlation coefficient of 0.996.^21^ Body water of 33% or less indicates dry skin, 34%–37% indicates mild dry skin, 38%–42% indicates general status, 43%–46% indicates mildly moist skin, and 47% or more indicates moist skin. When using the scanner, examiners had to confirm that it was fixed to the skin.

### 2.5 Statistical analysis

Data analysis was conducted using SPSS software (version 20.0; SPSS Inc., Chicago, IL, United States). Descriptive statistics, including means, SDs, and frequency distributions, were used to describe patient demographic data. Analysis of variance was performed to compare the confounders between groups. Cox regression was employed to compare the incidence density of HFSR and a generalised estimating equation was used to compare the percentage of cutaneous wetness between groups, factors, and multiple time points.

## 3 RESULTS

### 3.1 Comparison of confounders

As noted previously, 129 patients were randomly assigned to three groups. Table 1 presents the comparison of confounders between groups. No significant difference was found among the three groups.

**TABLE 1:** Comparison of confounders among groups

Variants	Comparison group (*n* = 43)	Moisturising cream (*n* = 43)	10% Urea‐based cream (*n* = 43)	*p*
Gender				.293
Male	32 (74.4%)	33 (76.7%)	36 (83.7%)	
Female	11 (25.6%)	10 (23.3%)	7 (16 .3%)	
Age	70.06 ± 9.95	71.18 ± 9.89	70.26 ± 7.98	.839
No of chronic illness	3.02 ± 1.08	2.93 ± 1.18	3.20 ± 1.35	.555
No of metastatic regions	0.60 ± 0.69	0.72 ± 0.73	0.63 ± 0.72	.730
Laboratory data				
WBC	6548 ± 3082	7935 ± 2490	6744 ± 2678	.085
Hb	12.10 ± 1.84	11.95 ± 1.54	12.15 ± 1.34	.859
PT	12.17 ± 1.48	12.46 ± 1.56	11.92 ± 1.48	.419
INR	1.18 ± 0.14	1.23 ± 0.20	1.19 ± 0.14	.407
Albumin	3.47 ± 0.46	3.53 ± 0.33	3.42 ± 0.49	.694
ALT	42.82 ± 14.79	39.68 ± 18.69	41.64 ± 18.32	.722
AST	42.45 ± 15.87	48.87 ± 20.96	39.93 ± 18.08	.831

Abbreviation: No, number.

### 3.2 Incidence density of HFSR

The incidence density of HFSR is presented in Table 2. Of the 125 patients, 56 (44.8%) developed HFSR. In the comparison group (BSC), 20 of 42 patients developed HFSR, with an incidence rate of 47.6% and incidence density of 1.12%. In group A (BSC plus moisturising cream), 18 of 41 patients developed HFSR, with an incidence rate of 43.9% and incidence density of 0.96%. In group B (BSC plus 10% urea‐based cream), 18 of 42 patients developed HFSR, with an incidence rate of 42.8% and incidence density of 0.92%.

**TABLE 2:** Incidence of density of hand‐foot skin reaction

Group	*N* (a)	HFSR (b)	Incidence rate (b/a)	Follow‐up days (c)	Incidence density (b/c)
Comparison	42	20	47.60%	1783	1.12%
Moisturising cream	41	18	43.90%	1877	0.96%
10% Urea‐based cream	42	18	42.80%	1966	0.92%

Abbreviation: HFSR, hand‐feet skin reaction.

A comparison of the incidence density of HFSR among the groups is presented in Table 3. No significant difference was observed between the comparison group and group A (hazard ratio in the comparison group was 1.19; 95% CI, 0.64–2.24, *p* > .05) as well as between the comparison group and group B (hazard ratio in comparison group was 1.033; 95% CI, 0.545–1.952, *p* > .05).

**TABLE 3:** Comparison of incidence density of hand‐foot skin reaction among groups

Variables	HR (95% CI)	*p*
Groups (Moisturising cream/comparison)	1.19(0.64 ~ 2.24)	.581
Groups (10% urea‐based cream/Comparison)	1.03(0.55 ~ 1.95)	.921

Abbreviation: HR, hazard ratio.

### 3.3 Comparisons of cutaneous wetness

**FIGURE 2:**
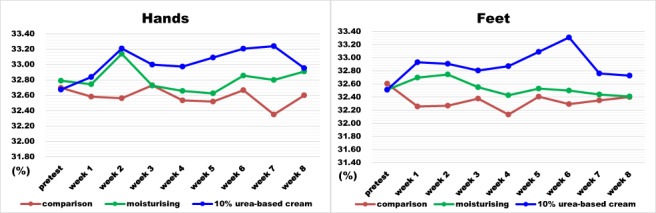
Changes in mean cutaneous wetness in hands and feet

The majority of patients had dry skin on their hands or feet at different time points. The cutaneous wetness was 32.35%–33.31% (as shown in Figure 2). The results indicated that BCS plus 10% urea‐based cream was the most effective in preventing sorafenib‐induced HFSR. Comparisons of cutaneous wetness in hands and feet among the groups are presented in Table 4. No significant differences were observed between the comparison group and group A as well as between the comparison group and group B in week 0 (*p* > .05). No significant difference was found between the varying time points of comparison groups (*p* > .05) as well as between the comparison group and group A (*p* > .05). Nevertheless, a significant difference was observed between the comparison group and group B after starting sorafenib. On the hands, group B exhibited better cutaneous wetness than the comparison group in week 7 (*p* < .05). On the feet, group B exhibited better cutaneous wetness than the comparison group in week 1 (*p* < .05), week 2 (*p* < .05), week 4 (*p* < .05), week 5 (*p* < .05), and week 6 (*p* < .001).

**TABLE 4:** Comparison of percentage of cutaneous wetness among groups

Parameter	Hands		Feet	
	Coefficient	*p*	Coefficient	*p*
Comparison group at week 0	32.7		32.6	
Week 0 (moisturising cream/comparison group)	0.1	.716	−0.1	.691
Week 0 (10% urea‐based cream/comparison group)	0.0	.927	−0.1	.691
Control group (week 1/week 0)	−0.1	.649	−0.3	.137
Control group (week 2/week 0)	−0.1	.597	−0.3	.156
Control group (week 3/week 0)	0.0	.904	−0.2	.353
Control group (week 4/week 0)	−0.2	.560	−0.5	.068
Control group (week 5/week 0)	−0.2	.538	−0.2	.460
Control group (week 6/week 0)	0.0	.918	−0.3	.258
Control group (week 7/week 0)	−0.3	.278	−0.3	.387
Control group (week 8/week 0)	−0.1	.761	−0.2	.486
Difference of slopes from week 0 to week 1 between groups (A/C)	0.1	.847	0.5	.107
Difference of slopes from week 0 to week 2 between groups (A/C)	0.5	.182	0.6	.088
Difference of slopes from week 0 to week 3 between groups (A/C)	−0.1	.793	0.3	.438
Difference of slopes from week 0 to week 4 between groups (A/C)	0.0	.937	0.4	.278
Difference of slopes from week 0 to week 5 between groups (A/C)	0.0	.973	0.2	.556
Difference of slopes from week 0 to week 6 between groups (A/C)	0.1	.815	0.3	.431
Difference of slopes from week 0 to week 7 between groups (A/C)	0.4	.415	0.2	.648
Difference of slopes from week 0 to week 8 between groups (A/C)	0.2	.628	0.1	.803
Difference of slopes from week 0 to week 1 between groups (B/C)	0.3	.440	0.8	.021^*^
Difference of slopes from week 0 to week 2 between groups (B/C)	0.7	.065	0.7	.028^*^
Difference of slopes from week 0 to week 3 between groups (B/C)	0.3	.428	0.5	.127
Difference of slopes from week 0 to week 4 between groups (B/C)	0.5	.228	0.8	.019^*^
Difference of slopes from week 0 to week 5 between groups (B/C)	0.6	.136	0.8	.034^*^
Difference of slopes from week 0 to week 6 between groups (B/C)	0.6	.175	1.1	.004^*^
Difference of slopes from week 0 to week 7 between groups (B/C)	0.9	.037^*^	0.5	.210
Difference of slopes from week 0 to week 8 between groups (B/C)	0.4	.397	0.4	.305

*Note*: A—group treated with a moisturising cream, B—group treated with a 10% urea‐based cream, C—comparison group.

^*^ *p* < .05.

## 4 DISCUSSION

The aim of this study was to validate the prophylactic efficacy of 10% urea‐based cream on sorafenib‐induced HFSR in patients with advanced HCC. The results indicated that BCS plus 10% urea‐based cream can significantly improve cutaneous wetness compared with BSC alone or BSC plus moisturising cream. However, no significant difference was observed in the incidence density of HFSR between the three groups.

The characteristics of patients were similar to the global distribution with a majority of elderly people and a gender ratio of 2–3 males to 1 female.^2^, ^6^ All followed the recommended guideline of taking oral sorafenib 400mg twice per day to treat advanced HCC with Child‐Pugh liver function class A.^3^, ^4^, ^7^ Compared with previous studies, the overall incidence of sorafenib‐related HFSR (44.8%) fell within the common range of an Asia‐Pacific population receiving standard treatment (10.7%–42%).^6^, ^11^ The three groups were recruited for a whole year, so cutaneous status influence with varied whether or daily events may cover. The majority of patients, who were elderly with dry or mild dry skin, were available during this time.

Ren et al. conducted a randomised controlled trial to validate the prophylactic efficacy between BSC with no creams and BSC with a 10% urea‐based cream for sorafenib‐induced HFRS in patients with advanced HCC.^17^ The results suggested that the cream significantly prolonged the median time of incidence. The discrepancy with the results of the current study may relate to an inadequate treatment dose and the pharmaceutical factory from which the cream was obtained. One previous study recommended applying a 10% urea‐based cream made in Germany three times a day after starting sorafenib on day 1.^17^ The current study followed the recommendations of the pharmaceutical factory in Taiwan, which was to apply the 10% urea‐based cream twice a day from 3 days prior to starting sorafenib. Thus, applying a nut‐sized amount of 10% urea‐based cream made by a Germany pharmaceutical factory three times a day may be more effective in preventing sorafenib‐induced HFSK. Additionally, this study also supported that an inadequate treatment dose or non‐Germany making of the 10% urea‐based cream may have an efficacy similar to that of the moisturising cream.

A randomised controlled phase II trial revealed that a hydrocolloid dressing containing ceramide fixed on soles every 2–3 days was significantly more effective in treating sorafenib‐induced grade 1 HFSR than the application of a 10% urea‐based cream 2–3 times a day in patients with renal cell carcinoma.^22^ Because it was not possible to apply hydrocolloid dressing over the hands, a moisturising cream was used as a substitute on the palms and soles in this study. No significant difference was found in incidence density on sorafenib‐induced HFSR, regardless of whether a moisturising cream or 10% urea‐based cream was applied. The moisturising cream was therefore not an effective substitute for the hydrocolloid dressing containing ceramide. Nevertheless, this study found that the 10% urea‐based cream was more effective in maintaining cutaneous wetness during the first 6 weeks of starting sorafenib. Painful hyperkeratosis was a common consequence that resulted in the disruption of treatment whether in clinic quo or in adherence to the recommended guidelines.^14^, ^15^, ^23^ Thus, the findings of this study indicate that a 10% urea‐based cream may prevent hyperkeratosis within the first 6 weeks of starting sorafenib, after which a moisturising cream can be applied. Although the prophylactic 10% urea‐based cream eventually maintained cutaneous wetness in dry or mild dry skin it was confidence to prevent worse tendency of it during treatment. Besides, using it twice a day merely maintain patients' cutaneous wetness below 34%, which cannot significantly prevent dry skin. The dose also cannot reduce incidence of HFSR. The evidence possibly indicated an adequate dose to the 10% urea‐based cream should be three times.

## 5 LIMITATION

Questions still remain as to the most appropriate treatment dose and dermatological agent. Future research should validate the efficacy of an increased treatment dose of urea‐based creams, such as comparing 10%, 12.5% or 20% as well as find a suitable substitute for hydrocolloid dressing containing ceramide, such as heparin cream that can be applied to the hands and feet.

## 6 CONCLUSION

In conclusion, the application of nut‐size amount of the 10% urea‐based cream twice a day may preserve cutaneous wetness in the first 6 weeks after starting sorafenib, but it cannot reduce the occurrence of HFSR. Thus, the result supports nut‐size dose of the 10% urea‐based cream three times a day may be an appropriate dose to prevent HFSR.

## CONFLICT OF INTEREST

The authors declared that they have no conflict of interest to this work.

## ETHICAL STATEMENTS

This study was approved by the Institutional Review Board of the Taipei Veterans General Hospital (2013‐13‐009B) and has been registered on website of ClinicalTrials.gov (NCT04568330). A case manager obtained informed consent from all patients prior to their participation in the study. The study protocol was explained to the patients and they were taught self‐care skills by the case manager. All were aware that their rights would be protected, that the risk of participation would be the lowest possible, and that the most suitable form of management would be arranged when they develop sorafenib‐induced HFSR.

## AUTHOR CONTRIBUTIONS

**R.‐Y.L.:** Conceptualization; data curation; formal analysis; investigation; methodology. **H.‐H.T.:** Conceptualization; methodology; writing – review and editing. **S.‐L.W.:** Conceptualization; data curation; formal analysis. **H.‐L.H.:** Conceptualization; writing – review and editing. **S.‐F.L.:** Data curation; investigation.

## Data Availability

Data of all collected participants was availability to share after deidentification.

## References

[1] Albreht T , Borras J , Conroy F . European Guide for Quality National Cancer Control Programs. Ljubljana: European Partnership for Action against Cancer; 2015.

[2] Bray F , Ferlay J , Soerjomataram I . Global cancer statistics 2018: GLOBOCAN estimates of incidence and mortality worldwide for 36 cancers in 185 countries. Cancer J Clin. 2018;68:394‐424. 10.3322/caac.21492 30207593

[3] European Association for the Study of the Liver . EASL clinical practice guidelines: management of hepatocellular carcinoma. J Hepatol. 2018;69:182‐236. 29628281 10.1016/j.jhep.2018.03.019

[4] Vogel A , Cervantes A , Chau I . Hepatocellular carcinoma: ESMO clinical practice guidelines for diagnosis, treatment and follow‐up. Ann Oncol. 2018;S4:iv238‐iv255. 10.1093/annonc/mdy308 30285213

[5] Llovet JM , Ricci S , Mazzaferro V . Sorafenib in advanced hepatocellular carcinoma. N Engl J Med. 2008;359:378‐390. 18650514 10.1056/NEJMoa0708857

[6] Cheng AL , Kang YK , Chen Z . Efficacy and safety of sorafenib in patients in the Asia‐Pacific region with advanced hepatocellular carcinoma: a phase III randomized double‐blind, placebo‐controlled trail. Lancet Oncol. 2009;10:25‐34. 19095497 10.1016/S1470-2045(08)70285-7

[7] National Comprehensive Cancer Network . NCCN clinical practice guidelines in oncology (NCCN Guidelines): hepatobiliary cancers. Accessed August 1, 2019. http://www.NCCN.org. 10.6004/jnccn.2009.0027 PMC4461147 19406039

[8] Cubero DIG , Abdalla BMZ , Schoueri J . Cutaneous side effect of molecularly targeted therapies for the treatment of solid tumors. Drug Context. 2018;7:212516. 10.7573/dic.212516 PMC6052912 30038659

[9] Lipworth AD , Robet C , Zhu AX . Hand‐foot syndrome (head‐foot skin reaction, palmar‐plantar erythrodysesthesia): focus on Sorafenib and Sunitinib. Oncology. 2009;77:257‐271. 19923864 10.1159/000258880

[10] Massey PR , Okman JS , Wilkerson J . Tyrosine kinase inhibitors directed against the vascular endothelial growth factor receptor (VEGFR) have distinct cutaneous toxicity profiles: a meta‐analysis and review of the literature. Support Care Cancer. 2015;23(6):1827‐1838. 25471178 10.1007/s00520-014-2520-9 PMC4414682

[11] Chang WT , Lu SN , Rau KM . Increase cumulative doses and appearance of hand‐foot skin reaction prolonged progression free survival in sorafenib‐treated advanced hepatocellular carcinoma patients. Kaohsiung J Med. 2018;34:391‐399. 10.1016/j.kjms.2018.03.006 PMC12977125 30063012

[12] Kao PH , Cen JS , Chung WH . Cutaneous adverse events of targeted anticancer therapy: a review of common clinical manifestations and management. J Cancer Res Pract. 2015;2(4):271‐284.

[13] Macdonald JB , Macdonald B , Golitz LE . Cutaneous adverse side effects of targeted therapy – part I: inhibitors of the cellular membrane. J Am Acad Dermatol. 2015;72(2):203‐218. 25592338 10.1016/j.jaad.2014.07.032

[14] Manchen E , Robert C , Porta C . Management of tyrosine kinase inhibitor‐induced hand‐foot skin reaction: viewpoints from the medical oncologist, dermatologist, and oncology nurse. J Support Oncol. 2011;9(1):13‐23. 21465734 10.1016/j.suponc.2010.12.007

[15] Wood LS , Lemont H , Jatoi A . Practical considerations in the management of hand‐foot skin reaction caused by multikinase inhibitors. Commun Oncol. 2010;7:23‐39.

[16] Celleno L . Topical urea in skincare: a review. Dermatol Ther. 2018;31:e12690. 30378232 10.1111/dth.12690

[17] Ren ZG , Zhu KS , Kng HY . Randomized controlled trial of the prophylactic effect of urea‐based cream on sorafenib‐associated hand‐foot skin reaction in patients with advanced hepatocellular carcinoma. J Clin Oncol. 2015;33:894‐900. 25667293 10.1200/JCO.2013.52.9651

[18] Negri FV . Urea‐based cream to prevent sorafenib‐induced hand‐and‐foot skin reaction: which evidence? J Clin Oncol. 2015;33(28):3219. 26215963 10.1200/JCO.2015.61.6417

[19] Faul F , Erdfelder E , Bunchner A , Lang AG . Statistical power analysis using G*power 3.1: test for correlation and regression analysis. Behav Res Methods. 2009;41(4):1149‐1160. 19897823 10.3758/BRM.41.4.1149

[20] Dueck AC , Mendoza TR , Mitchell SA . Validity and reliability of the US National Cancer Institute's patient‐reported outcomes version of the common terminology criteria for adverse events (PRO‐CTAE). JAMA Oncol. 2015;1(8):1051‐1059. 26270597 10.1001/jamaoncol.2015.2639 PMC4857599

[21] Piccoli A , Rossi B , Pillon L , Bucciante G . A new method for monitoring body fluid variation by bioimpedance analysis: the RXc graph. Kidney Int. 1994;46:534‐539. 7967368 10.1038/ki.1994.305

[22] Shinohara N , Nonmura N , Eto M . A randomized multicenter phase II trial on the efficacy of a hydrocolloid dressing contain ceramide with a low‐friction external surface for hand‐foot skin reaction caused by sorafenib in patients with renal cell carcinoma. Ann Oncol. 2014;25:472‐476. 24351402 10.1093/annonc/mdt541

[23] Barton‐Burke M , Ciccolini K , Mekas M . Dermatologic reactions to targeted therapy: a focus on epidermal growth factor receptor inhibitors and nursing care. Nurs Clin North Am. 2017;52(1):83‐113. 28189168 10.1016/j.cnur.2016.11.005 PMC5645079

